# Demographic Characteristics, Clinical Features, and Treatment Patterns of Classic and AIDS-Related Kaposi Sarcoma: A Multicenter Real-World Study from Turkey

**DOI:** 10.3390/medicina62081483

**Published:** 2026-08-01

**Authors:** Ahmet Kürşad Dişli, Bekir Mert Durukan, Esra Aşık, Ayşe Nuransoy Cengiz, Gamze Emin, Hasan Çağrı Yıldırım, Şafak Yıldırım Dişli, Deniz Can Güven, Serkan Akın, Oktay Bozkurt, Feyyaz Özdemir, Mevlüde İnanç, Metin Özkan

**Affiliations:** 1 Department of Medical Oncology, Kırşehir Education and Research Hospital, 40100 Kırşehir, Turkey; 2 Department of Medical Oncology, Faculty of Medicine, Hacettepe University, 06230 Ankara, Turkey; 3 Department of Medical Oncology, Faculty of Medicine, Karadeniz Technical University, 61080 Trabzon, Turkey; 4 Department of Medical Oncology, Faculty of Medicine, Erciyes University, 38039 Kayseri, Turkey; 5 Department of Medical Oncology, Faculty of Medicine, Ege University, 35100 İzmir, Turkey; 6 Department of Medical Oncology, Acıbadem Kayseri Hospital, 38140 Kayseri, Turkey

**Keywords:** Kaposi sarcoma, classic Kaposi sarcoma, AIDS-related Kaposi sarcoma, real-world evidence, multicenter cohort, Turkey

## Abstract

*Background and Objectives:* Kaposi sarcoma (KS) is a rare angioproliferative malignancy driven by human herpesvirus-8 infection. Comparative real-world data on classic and AIDS-related KS from Mediterranean-endemic countries remain limited. *Materials and Methods:* We retrospectively analyzed 102 patients with histopathologically confirmed KS treated across five tertiary oncology centers in Turkey between January 2010 and December 2023. Demographic characteristics, clinical features, treatment patterns, and survival outcomes were compared between classic and AIDS-related KS subtypes. *Results:* Ninety-two patients (90.2%) had classic KS and ten (9.8%) had AIDS-related KS. The median age at diagnosis was significantly higher in the classic KS group (69 vs. 38.5 years; *p* < 0.001). Male sex predominated in both groups (classic KS: 76.1%; AIDS-related KS: 100%; *p* = 0.113). AIDS-related KS presented with more advanced disease at diagnosis, with 70% classified as poor-risk by ACTG-TIS criteria versus 9.8% Stage IV by Brambilla criteria in classic KS, and more frequent extracutaneous involvement (70% vs. 8.7%; *p* < 0.001). Paclitaxel was the most commonly used first-line agent in classic KS (42.9%), while pegylated liposomal doxorubicin predominated in AIDS-related KS (87.5%). Overall response rates were high and comparable between groups (90.7% vs. 88.9%). Median overall survival was significantly longer in classic KS (66.9 vs. 22.67 months; *p* = 0.012). Disease stage and lesion morphology were not significantly associated with survival in either group. *Conclusions:* Classic and AIDS-related KS differ substantially in clinical presentation and survival outcomes, yet demonstrate comparable chemosensitivity. Our findings in the AIDS-related Kaposi sarcoma group are preliminary and hypothesis-generating due to the small sample size, requiring confirmation in larger prospective cohorts.

## 1. Introduction

Kaposi sarcoma (KS) is a rare angioproliferative neoplasm of endothelial origin, first described by the Hungarian dermatologist Moritz Kaposi in 1872 as “idiopathic multiple pigmented sarcoma of the skin.” The disease is etiologically linked to Kaposi sarcoma-associated herpesvirus, also known as human herpesvirus-8 (KSHV/HHV-8), a member of the Gammaherpesvirinae subfamily [1]. According to the World Health Organization, KS is defined as a locally aggressive endothelial proliferation that typically manifests as multiple patches, plaques, or nodules on the skin, with the potential to involve mucosal surfaces, lymph nodes, and visceral organs [2]. Clinical recognition of KS relies primarily on its characteristic morphological features; dermoscopic evaluation has further emerged as a valuable non-invasive adjunct in this context. The rainbow pattern—a polychromatic dermoscopic appearance arising from the dispersion of polarized light through vascular tissue components—has been established as a hallmark, albeit not entirely pathognomonic, feature of cutaneous KS [3].

Four distinct epidemiological subtypes have been recognized: classic (Mediterranean/sporadic) KS, epidemic (AIDS-related) KS, endemic (African) KS, and iatrogenic (immunosuppression-related) KS [4]. Although these subtypes share common histopathological features, they differ substantially in terms of patient demographics, clinical behavior, and prognosis. Classic KS predominantly affects elderly men of Mediterranean and Eastern European descent, typically follows an indolent course, and is largely confined to the skin of the lower extremities [5,6]. In contrast, AIDS-related KS tends to occur in younger individuals with profound immunosuppression, presents with more widespread cutaneous and extracutaneous involvement, and carries a more aggressive clinical trajectory [7].

The development of KS requires not only KSHV infection but also the co-occurrence of genetic susceptibility, immune dysregulation, and environmental cofactors [8]. Once latent infection is established, KSHV encodes a repertoire of oncogenic proteins—including LANA-1, v-cyclin, and viral FLICE inhibitory protein—that collectively drive apoptosis resistance, cell proliferation, angiogenesis, inflammation, and immune evasion [9,10]. These mechanisms underpin both the multifocal nature of the disease and its notable sensitivity to immunological restoration, as evidenced by tumor regression following antiretroviral therapy in HIV-positive patients or modification of immunosuppressive regimens in transplant recipients [11].

Geographically, KS incidence is highest in sub-Saharan Africa and Mediterranean countries, where KSHV seroprevalence rates are considerably elevated [5]. Turkey, occupying a central position within the Mediterranean basin, represents an endemic area for classic KS, and several single-center series from Turkish institutions have made valuable contributions to the real-world understanding of this disease [12,13,14]. These studies have consistently demonstrated that classic KS in Turkey predominantly affects older patients, with lower extremity involvement being the most common presentation, and that paclitaxel and pegylated liposomal doxorubicin (PLD) remain the most frequently employed systemic agents in advanced disease [13,14]. Building upon this growing body of evidence, multicenter data encompassing both classic and AIDS-related subtypes from geographically diverse centers across Turkey could further enrich our understanding of disease heterogeneity and treatment patterns at a national level.

To address this, we conducted a multicenter retrospective study across five tertiary oncology centers in Turkey. Our primary objectives were to characterize the demographic and clinical features of patients with classic and AIDS-related KS, to describe real-world treatment patterns including first- and second-line chemotherapy choices, and to compare clinical outcomes between the two subtypes. By pooling data from geographically diverse centers, this study aims to provide a more comprehensive picture of KS management in Turkey and to contribute to the growing literature on this rare malignancy.

## 2. Materials and Methods

### 2.1. Study Design and Patient Population

This multicenter retrospective cohort study was conducted across five tertiary oncology centers in Turkey: Erciyes University Faculty of Medicine (Kayseri), Hacettepe University Faculty of Medicine (Ankara), Karadeniz Technical University Faculty of Medicine (Trabzon), Ege University Faculty of Medicine (Izmir), and Acıbadem Kayseri Hospital (Kayseri). Medical records of patients with histopathologically confirmed KS who were followed between January 2010 and December 2023 were reviewed. Patients aged 18 years or older with a histologically proven diagnosis of KS were eligible for inclusion. Those with incomplete medical records precluding the extraction of essential clinical data and misdiagnosis were excluded. A total of 102 patients were included in the final analysis, comprising 92 with classic KS and 10 with AIDS-related KS.

### 2.2. Data Collection

Baseline demographic, clinical, and laboratory data were retrieved from institutional electronic medical records. Recorded variables included age at diagnosis, sex, ECOG performance status, comorbid conditions, HIV serostatus, prior immunosuppressive therapy, and history of solid organ transplantation. Disease-related variables encompassed lesion number and anatomical distribution, clinical morphology, presence of symptoms, extracutaneous involvement, time from symptom onset to diagnosis, disease stage at presentation, and HHV-8 PCR status. However, antiretroviral therapy status at KS diagnosis, timing of ART relative to chemotherapy initiation, and longitudinal CD4 count data could not be reliably and completely reconstructed from retrospective records and were therefore not included in the analysis.

### 2.3. Disease Staging

Disease extent was classified according to the staging system proposed by Brambilla et al. for classic KS. For AIDS-related KS, the AIDS Clinical Trials Group (ACTG) staging system was applied where applicable.

### 2.4. Treatment and Response Assessment

Systemic treatment indications, chemotherapy regimens administered in the first- and second-line settings, and best treatment responses were recorded for all patients who received systemic therapy. Because Kaposi’s sarcoma involves multifocal lesions not consistently measurable by standard imaging, response assessment followed a KS-adapted clinical framework rather than RECIST 1.1 or WHO criteria. Complete response (CR) was defined as the total disappearance of all clinically detectable lesions. Partial response (PR) was defined as a reduction of at least 25% in the number or size of lesions in the absence of new lesions. Progressive disease (PD) was defined as the appearance of new lesions or an increase of 25% or greater in existing lesions. This ≥25% threshold deviates from standard RECIST 1.1 and WHO response criteria, and should be interpreted in the context of KS-specific response assessment. Responses not fulfilling these criteria were classified as stable disease (SD). The objective response rate (ORR) was defined as the proportion of patients achieving CR or PR, and the disease control rate (DCR) as the proportion achieving CR, PR, or SD. Because response data were ascertained retrospectively from heterogeneous clinical records, treatment-specific response rates are reported descriptively and were not formally compared between regimens.

### 2.5. Survival Analysis

Overall survival (OS) was calculated from the date of diagnosis to the date of death from any cause or last follow-up. Progression-free survival (PFS) was defined as the time from initiation of first-line systemic therapy to disease progression or death from any cause. Patients alive without disease progression at the time of last follow-up were censored. Survival curves were estimated using the Kaplan–Meier method, and differences between groups were evaluated with the log-rank test.

### 2.6. Statistical Analysis

Normality of continuous variables was assessed using the Kolmogorov–Smirnov test with Lilliefors correction for groups with *n* > 50 [15] and the Shapiro–Wilk test for groups with *n* < 50 [16]. Normally distributed variables were compared between groups using the independent samples t-test and are presented as mean ± standard deviation. Non-normally distributed variables were compared using the Mann–Whitney U test and are presented as median with minimum-maximum range. Categorical variables were compared using Fisher’s exact test, applied in all instances where expected cell counts fell below five [17], and are presented as frequencies and percentages. Pairwise comparisons of proportions were performed using Bonferroni-corrected Z-tests, and results are reported using letter-based notation. The association between clinical characteristics and progression-free or overall survival was examined using the Kaplan–Meier method. A two-sided *p*-value of less than 0.05 was considered statistically significant. All analyses were performed using IBM SPSS Statistics, version 26 (IBM Corp., Armonk, NY, USA) [18].

### 2.7. Ethics

This study was conducted in accordance with the ethical principles outlined in the Declaration of Helsinki. Ethical approval was obtained from the Medical Research Institutional Review Board of Acıbadem University (approval number: 2026-03/28, date: 5 February 2026). Informed consent was waived owing to the retrospective nature of the study.

## 3. Results

### 3.1. Patient Characteristics and Clinical Features

**Figure 1:**
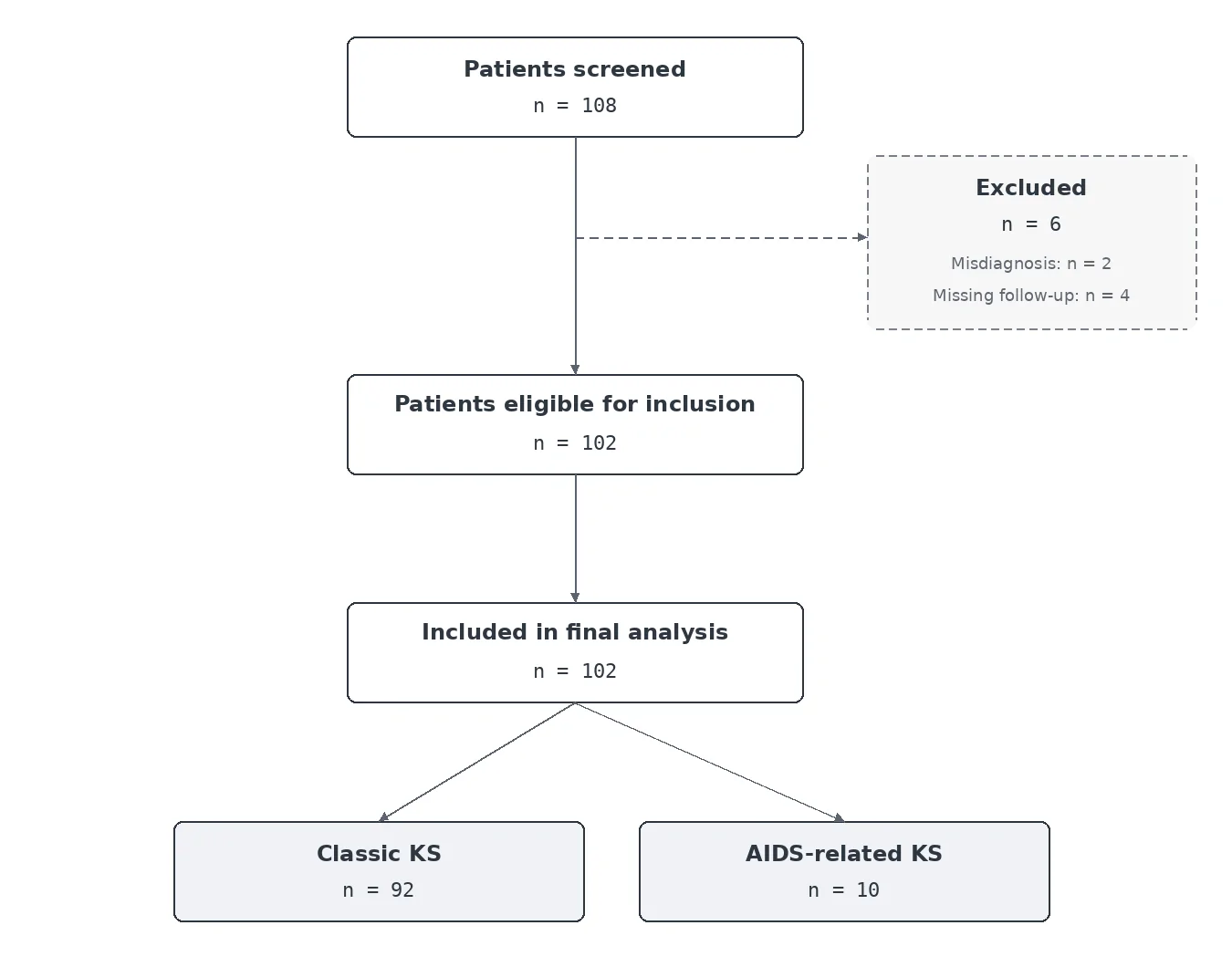
Patient flow diagram (STROBE).

A total of 108 patients with a diagnosis of Kaposi’s sarcoma were initially screened. Six patients were excluded: 2 due to misdiagnosis and 4 due to missing follow-up data. (Figure 1) A total of 102 patients with histopathologically confirmed KS were included, of whom 92 (90.2%) had classic KS and 10 (9.8%) had AIDS-related KS. Baseline demographic and clinical characteristics are summarized in Table 1. The median age at diagnosis was significantly higher in the classic KS group compared with the AIDS-related group (69 years [range, 19–89] vs. 38.5 years [range, 23–64]; *p* < 0.001). Male sex predominated in the classic KS group (76.1%), whereas all patients in the AIDS-related KS group were male (100%; *p* = 0.113). The majority of patients in both groups had an ECOG performance status of 0 or 1 (classic: 93.5%; AIDS-related: 100%; *p* = 0.344).

The number of lesions at presentation differed significantly between groups (*p* = 0.004). In the classic KS group, 78.3% of patients had fewer than 10 lesions, whereas 60% of AIDS-related KS patients presented with 10 to 100 lesions. Disease localization differed markedly between subtypes (*p* < 0.001), with lower extremity involvement predominating in classic KS (66.3%) and multifocal distribution being the most common pattern in AIDS-related KS (60%). Head and neck involvement was more frequent in the AIDS-related group (20% vs. 1.1%). Plaque was the most common lesion morphology in both groups (classic: 43.2%; AIDS-related: 44.4%; *p* = 0.549). Extracutaneous involvement was significantly more frequent in the AIDS-related KS group (70% vs. 8.7%; *p* < 0.001). The rates of symptomatic disease, painful lesions, lymphedema, ulceration, and time from symptom onset to diagnosis were comparable between groups (all *p* > 0.05). In the AIDS-related KS group, 3 patients (30%) were classified into the good risk category, whereas 7 patients (70%) were in the poor risk category. In contrast, within the classic KS group, 79 patients (85.9%) were classified as Stage I-II, 4 (4.3%) as Stage III, and 9 (9.8%) as Stage IV. Since distinct staging systems (Brambilla and ACTG-TIS) were utilized for the classic and AIDS-related KS subtypes, stage distributions are presented solely for descriptive purposes within their respective subgroups.

However, the highly limited number of patients in the AIDS-related KS group (*n* = 10) mandates a cautious interpretation of the findings regarding this subgroup. Therefore, the results obtained should not be viewed as definitive conclusions, but rather as preliminary and hypothesis-generating findings for future larger-scale studies.

### 3.2. Treatment Patterns

Treatment modalities are presented in Table 2. In the classic KS group, radiotherapy or cryotherapy was the most frequently employed initial treatment (34.8%), followed by surgical excision (25%) and systemic chemotherapy (20.7%). In the AIDS-related KS group, systemic chemotherapy was the predominant treatment modality (70%). Systemic therapy was administered to 39.3% of classic KS patients and 80% of AIDS-related KS patients. Among patients receiving first-line chemotherapy, paclitaxel was the most commonly used agent in the classic KS group (42.9%), while PLD predominated in the AIDS-related group (87.5%). Local recurrence or metastatic disease occurred in 54.5% of classic KS patients and 66.7% of AIDS-related KS patients. For recurrent disease, radiotherapy was the most frequently used treatment in the classic KS group (62.8%), while chemotherapy predominated in the AIDS-related group (60%). In the second-line setting, PLD and oral etoposide were each administered in 33.3% of classic KS patients, while PLD and paclitaxel were each used in 50% of AIDS-related KS patients.

Table 2 represents the best clinical response achieved following any first-line treatment administered, including surgical excision, radiotherapy, cryotherapy, chemotherapy, and combined treatment approaches. These response rates should not be utilized to evaluate treatment-specific efficacy or chemotherapy sensitivity; rather, they should be interpreted as overall clinical response rates in which all treatment modalities are evaluated collectively.

### 3.3. Treatment Responses

The treatment responses and chemotherapy (CT) regimens for both cohorts are detailed in Table 3. In the classic patient group, one patient achieved an SD response with first-line therapy while receiving liposomal doxorubicin. Among patients in this group who achieved a PR or CR, paclitaxel was the most frequently administered CT, accounting for 40% and 41.2% of the cases, respectively. Two patients in the classic group exhibited PD, both of whom were treated with paclitaxel; overall, a 38.5% majority of the patients who experienced disease progression in this cohort were receiving paclitaxel. Regarding the epidemic (AIDS-related) group, Table 3 outlines that two patients achieved a first-line PR (one on liposomal doxorubicin and one on paclitaxel). A first-line CR was documented in six epidemic (AIDS-related) patients, all of whom (100%) received liposomal doxorubicin CT. Finally, disease progression occurred in only two patients within this subgroup, with one receiving liposomal doxorubicin and the other paclitaxel.

The treatment response rates reported in our study were obtained by collectively evaluating heterogeneous treatment modalities, including surgical excision, radiotherapy, cryotherapy, chemotherapy, and combined treatment approaches. Therefore, the reported response rates do not reflect treatment-specific efficacy or chemotherapy sensitivity, and should be regarded as a descriptive indicator of overall clinical response. Furthermore, subgroup comparisons by treatment modality were not performed in the AIDS-related KS group due to the limited number of patients.

### 3.4. Survival Analysis

The Kaplan–Meier analysis displays the unadjusted survival comparisons between groups, whereas the independent effects of other clinical variables that could influence survival were separately evaluated using Cox regression analysis.

**Figure 2:**
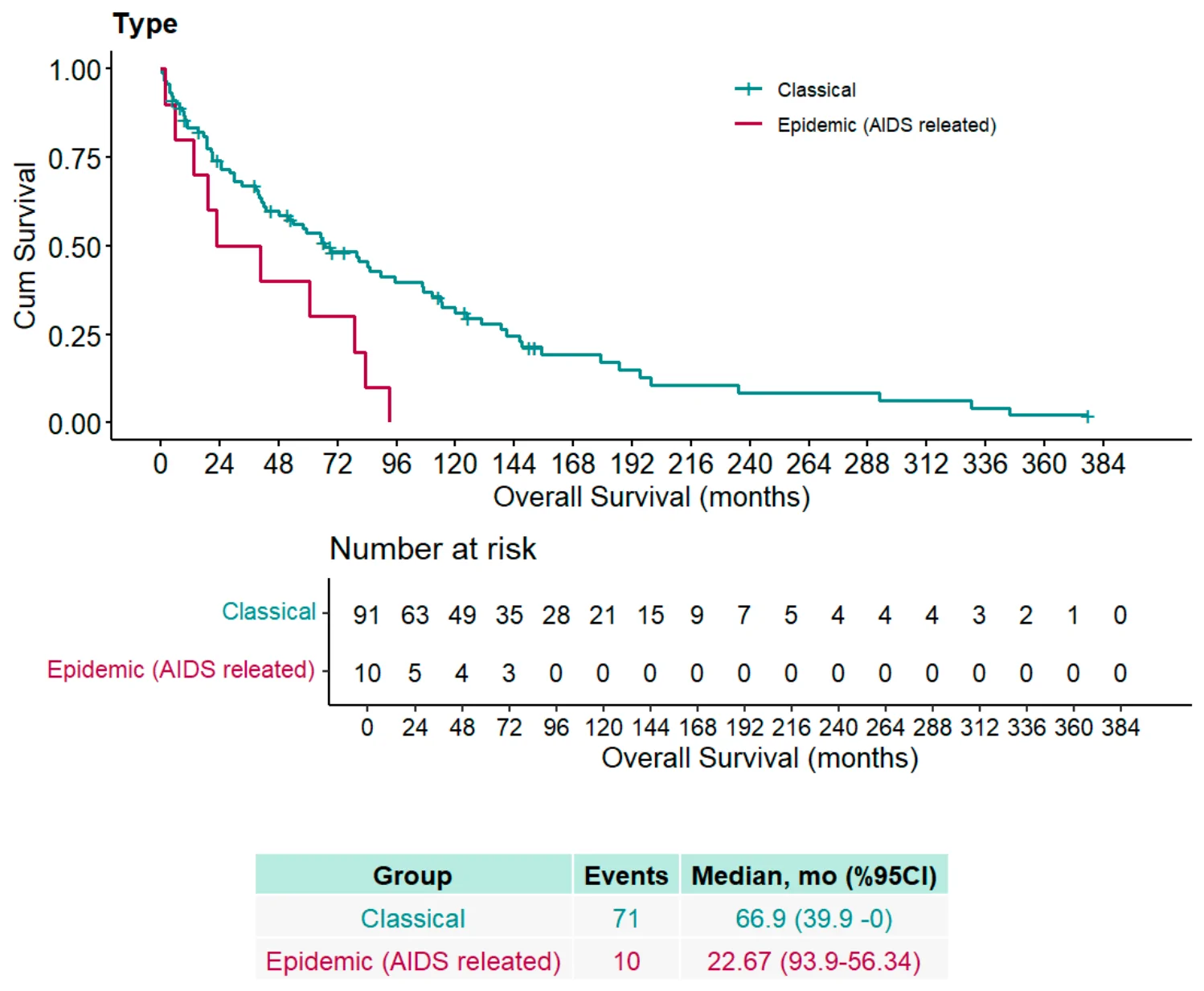
Kaplan–Meier overall survival curves by Kaposi sarcoma subtype (classic vs. AIDS-related).

**Figure 3:**
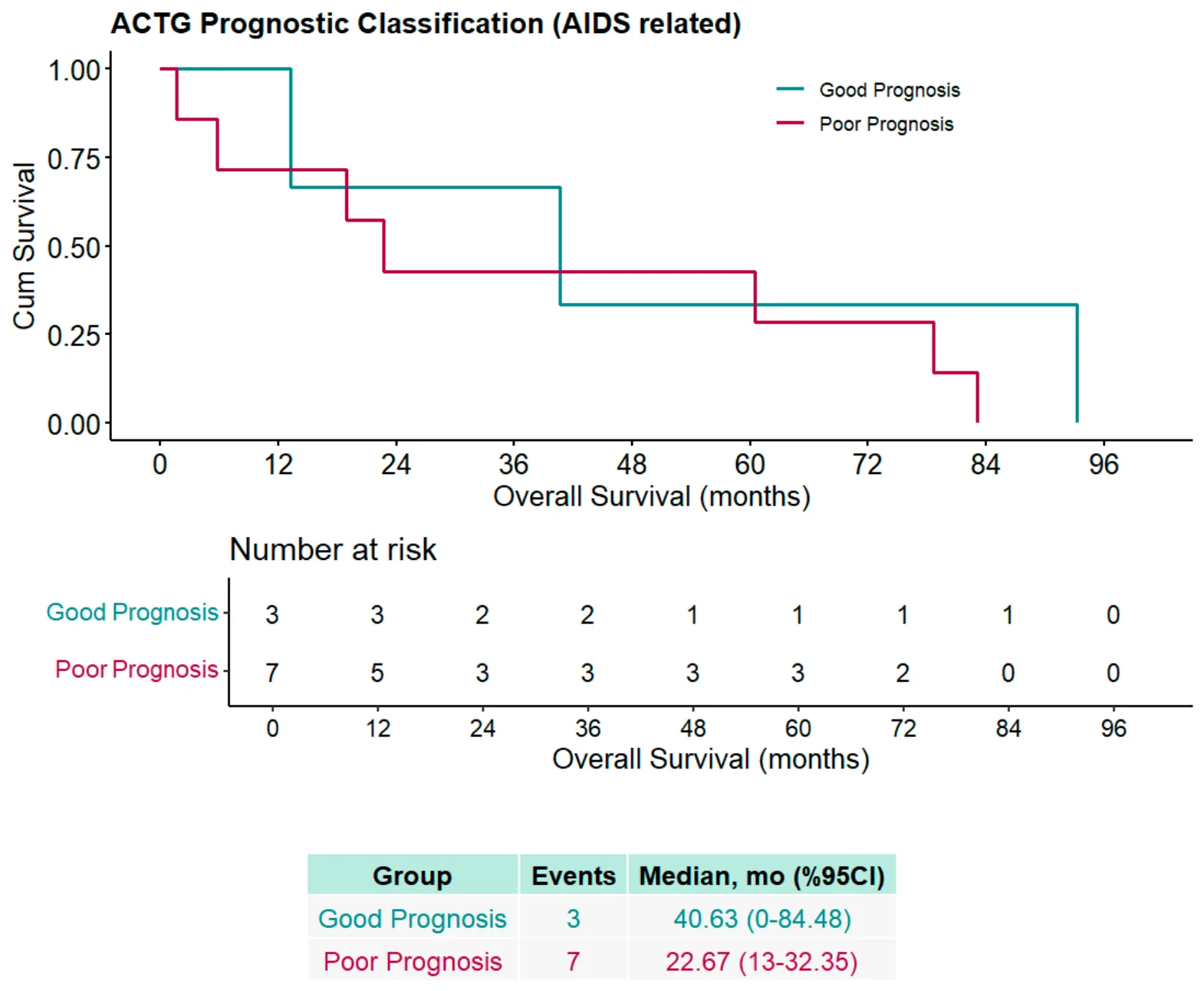
Kaplan–Meier overall survival curves by AIDS-related Kaposi sarcoma.

**Figure 4:**
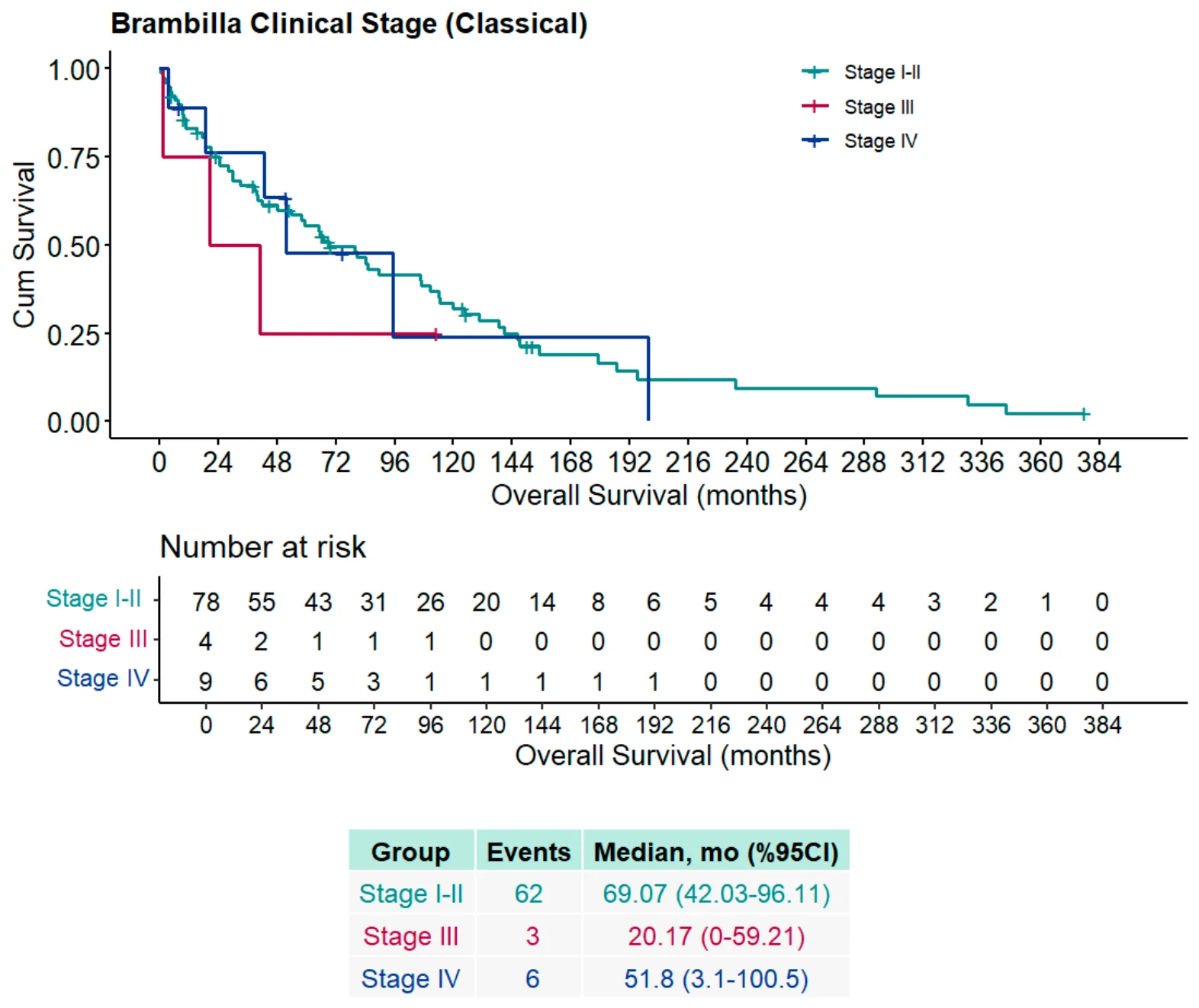
Kaplan–Meier overall survival curves by disease stage at diagnosis (Stages I-II, Stage III, Stage IV).

Kaplan–Meier survival estimates are presented in Table 4 and illustrated in Figure 2, Figure 3 and Figure 4. Overall survival differed significantly between subtypes (*p* = 0.012), with a median OS of 66.9 months (95% CI: 39.9–93.9) in the classic KS group and 22.67 months (95% CI: 0–56.34) in the AIDS-related KS group. Neither clinical lesion morphology (*p* = 0.373) nor disease stage at diagnosis (*p* = 0.302) was significantly associated with overall survival. Progression-free survival did not differ significantly between subtypes in either the first-line (*p* = 0.982) or second-line (*p* = 0.627) settings, nor was PFS significantly associated with clinical form or disease stage in either treatment line (all *p* > 0.05).

Based on the Kaplan–Meier survival analysis, 81 deaths (80.2%) occurred during the follow-up period, while 20 patients (19.8%) were censored. The median overall survival time was calculated as 65.13 months (95% CI: 41.07–89.19). It was observed that approximately half of the patients passed away within 65 months, whereas the remaining half survived longer than this duration.

### 3.5. Cox Regression Analysis

Cox regression analyses were performed to identify prognostic factors affecting overall survival in the classic group. When evaluating the analysis results, the 95% confidence intervals for some variables were found to be wide. The primary reason for this is the limited number of patients and events in certain subcategories. In particular, the low number of observations in the ECOG ≥2 group, certain clinical form subgroups, and some categorical variables reduced the stability of hazard ratio estimates, leading to wider confidence intervals. Therefore, results for variables with wide confidence intervals should be interpreted with caution, keeping in mind that the estimates carry statistical uncertainty.

The majority of clinical and laboratory variables examined in the univariate analysis were not found to be significantly associated with overall survival. Regarding age at diagnosis, although an increasing trend in the risk of death was observed with advancing age, this association did not reach statistical significance (HR = 1.018, 95% CI: 0.998–1.038, *p* = 0.076). ECOG performance status, the presence of comorbidity, smoking status, number of lesions, clinical form, presence of symptoms, painful lesion, lymphedema, ulceration, and extracutaneous involvement did not have a significant effect on overall survival (*p* > 0.05). Among laboratory parameters, a significant association was found between hemoglobin level and overall survival. Each 1-unit increase in hemoglobin level was found to reduce the risk of death by approximately 14.1% (HR = 0.859, 95% CI: 0.750–0.984, *p* = 0.028). Similarly, LDH level was also significantly associated with overall survival (HR = 0.993, 95% CI: 0.988–0.998, *p* = 0.003). In contrast, neutrophil, lymphocyte, monocyte, platelet, CRP, and albumin levels did not have a significant effect on overall survival (*p* > 0.05). Based on the univariate analysis results, age, ECOG performance status, smoking history, clinical form, hemoglobin, and LDH were included in the multivariate model.

In the multivariate analysis, the model fit was found to be statistically significant (χ^2^ = 20.710, *p* = 0.014). Age, ECOG performance status, smoking history, clinical form, hemoglobin level, and LDH level were evaluated together in the model. The analysis identified hemoglobin and LDH levels as independent prognostic factors for overall survival. Each 1-unit increase in hemoglobin level was found to reduce the risk of death by approximately 22.8% (HR = 0.772, 95% CI: 0.636–0.936, *p* = 0.009). This finding indicates that lower hemoglobin levels are associated with poorer overall survival. Similarly, LDH level was also independently associated with overall survival (HR = 0.988, 95% CI: 0.980–0.996, *p* = 0.003). Age, ECOG performance status, smoking status, and clinical form did not achieve statistical significance in the multivariate analysis (*p* > 0.05). However, the ECOG 1 category showed a trend toward a lower risk of death compared to the reference group, although this association did not reach the threshold of statistical significance (HR = 0.357, 95% CI: 0.116–1.098, *p* = 0.072). Overall, hemoglobin and LDH levels appear to be the primary prognostic markers independently affecting overall survival in the classic group, independent of other clinical variables (Table 5).

## 4. Discussion

This multicenter retrospective cohort study analyzed 102 patients with classic and AIDS-related KS managed across five tertiary oncology centers in Turkey. Our findings contribute real-world data on clinical presentation, treatment selection patterns, and survival outcomes to a literature that remains predominantly composed of small single-institution series, many of which focus exclusively on a single KS subtype.

The median age at diagnosis in the classic KS group was 69 years, consistent with the well-established epidemiological profile of this subtype and in keeping with prior Turkish and international series reporting median ages between 65 and 71 years [6,11,13]. AIDS-related KS patients were significantly younger, with a median age of 38.5 years, reflecting the disease’s predilection for younger immunocompromised individuals and mirroring findings from comparable published cohorts [7,12]. The male predominance observed in the classic KS group (76.1%) is consistent with the well-established epidemiological profile of this subtype, with reported male-to-female ratios ranging from 3:1 to 17:1 across published series [4,19]. All patients in the AIDS-related KS group were male, reflecting the predominance of this subtype among men who have sex with men and individuals with HIV infection [7].

The clinical presentations of both subtypes were consistent with their recognized phenotypes and aligned with findings from comparable series. Classic KS presented predominantly as early-stage, localized disease involving the lower extremities (66.3%), with limited extracutaneous spread (8.7%), mirroring the indolent clinical course characterizing this subtype in Mediterranean populations [5,6,11]. AIDS-related KS, by contrast, manifested with markedly advanced disease at diagnosis—70% of patients classified as poor-risk by ACTG-TIS criteria—widespread multifocal anatomical distribution (60%), and a substantially higher rate of extracutaneous involvement (70% vs. 8.7%; *p* < 0.001). This dichotomy reflects the fundamentally different immunological substrates of the two subtypes: while classic KS arises against a background of age-related immunosenescence and typically remains cutaneous for years, AIDS-related KS emerges in the setting of profound CD4 lymphopenia—median CD4 count of 11 cells/mm3 in our cohort—enabling aggressive endothelial proliferation and systemic dissemination [1,7]. Aydin et al. similarly reported markedly higher rates of lymph node and mucosal involvement in AIDS-related KS compared with the classic form within a Turkish cohort [7], and a large Italian multicenter series identified extracutaneous spread as the primary discriminating clinical feature between KS subtypes [19].

Treatment selection patterns in our cohort reflect institutional experience and patient profile. Among patients with classic KS, local therapies—predominantly radiotherapy and cryotherapy—constituted the initial treatment modality in 34.8% of patients, consistent with guideline recommendations for limited symptomatic disease [5]. KS has long been recognized as a highly radiosensitive tumor, and a recent retrospective analysis demonstrated a 100% overall response rate with radiotherapy for classic KS skin lesions, with complete response in 92.8% of treated sites, reinforcing its role as an effective and well-tolerated locoregional option [20]. Among patients requiring systemic therapy, paclitaxel was the most frequently used first-line agent in the classic KS group (42.9%), while PLD predominated in the AIDS-related group (87.5%). This treatment pattern is consistent with that reported by other Turkish centers [12,13,14]. Although international guidelines recommend PLD as the preferred first-line agent, paclitaxel remains a well-established and extensively validated option in KS [5,21] and represents a particularly valuable alternative in elderly classic KS patients in whom cardiac comorbidities may preclude long-term PLD use [13].

Response rates to initial treatment were high and comparable between subtypes (classic: 90.7%; AIDS-related: 88.9%), reflecting the well-established chemosensitivity of KS regardless of subtype [5,19]. Among patients receiving first-line systemic chemotherapy, the CR rate with paclitaxel in the classic KS group was 48.6%, consistent with the 79.6% ORR and 35.1-month median PFS reported by Paksoy et al. in a dedicated paclitaxel series for classic KS [13] and with the 68.7% ORR documented in a recent real-world comparative analysis of paclitaxel versus PLD [22]. In the AIDS-related group, all complete responses were achieved with PLD, yielding a CR rate of 75%, consistent with the established evidence base for PLD in this subtype: Cianfrocca et al. reported a 56% response rate for PLD in a randomized trial of AIDS-related KS, noting a more favorable toxicity profile compared with paclitaxel [23]. In the second-line setting, a PR rate of 58.3% in the classic KS group is broadly consistent with the 75% ORR reported by Khanmammadov et al. for second-line paclitaxel and etoposide in classic and iatrogenic KS [14], suggesting that salvage systemic options remain clinically meaningful beyond first-line failure. The inferior second-line outcomes in the AIDS-related group likely reflect the profoundly advanced immunosuppression and high disease burden in this subgroup rather than intrinsic drug resistance, as CD4 recovery through effective antiretroviral therapy is a critical determinant of long-term disease control in HIV-associated KS [24].

Local recurrence or metastatic progression occurred in more than half of patients in both groups (classic: 54.5%; AIDS-related: 66.7%), underscoring the chronic and relapsing nature of KS independent of subtype. This high recurrence rate is consistent with published series: Russo et al. documented a 22% relapse rate over a median follow-up of 5.8 years in a multicenter Italian cohort, with a median interval to recurrence of 2.7 years [19], and Kavak and Urun reported local recurrence in 63.2% of classic KS patients during a median follow-up of 69 months [6]. These persistently high recurrence rates across both subtypes emphasize the importance of sustained long-term follow-up and individualized retreatment strategies, as repeated cycles of local or systemic therapy are often required to maintain symptom control over the prolonged disease course characteristic of KS [5,21].

Overall survival differed significantly between subtypes (*p* = 0.012), with a median OS of 66.9 months in the classic KS group and 22.67 months in the AIDS-related group. The classic KS survival data in our cohort are broadly comparable to those reported in prior Turkish series—Oyucu Orhan et al. reported a median OS of 66.1 months in a mixed KS cohort [12]—though lower than population-based Italian registry data in which the median survival of classic KS patients approximated 9.4 years, not significantly different from the age-matched general population [25]. This divergence likely reflects the higher comorbidity burden, more heterogeneous treatment approach, and older age at presentation in our cohort. For AIDS-related KS, the inferior OS is attributable to the severity of underlying immunosuppression and the predominance of advanced-stage disease at presentation rather than differential chemosensitivity per se. The 2025 WHO guidelines for HIV-associated KS explicitly recommend immediate ART initiation combined with systemic chemotherapy in patients with severe symptomatic disease, recognizing that immune reconstitution synergizes with cytotoxic therapy to achieve durable disease control [24]. The markedly low CD4 counts in our AIDS-related cohort indicate that many patients were presenting with severely decompensated HIV disease, which likely compounded the prognostic impact of KS itself.

Neither disease stage nor clinical lesion morphology was significantly associated with overall survival in our cohort. This finding diverges from stage-dependent survival gradients reported in other series—including Kavak and Urun, who documented a significant OS difference between localized, locally advanced, and metastatic disease categories (*p* = 0.005) [6]—but is consistent with Hafizoglu et al., in whose 28-year cohort only ECOG performance status emerged as an independent predictor of OS on multivariate analysis [11]. A landmark prognostic factor analysis of 248 classic KS patients by Brenner et al. similarly identified immunosuppression and older age rather than anatomical disease stage as the strongest independent predictors of disease progression and dissemination, suggesting that host factors may carry greater prognostic weight than disease distribution in this malignancy [26]. The absence of a significant relationship between stage and survival in our study may additionally reflect the limited number of events within each staging subgroup, the heterogeneous treatment approaches inherent to a multicenter design, and the confounding effect of non-KS-related mortality. The inability to perform multivariate Cox regression analysis, precluded by sample size constraints especially in the AIDS-related subgroup, represents a significant methodological limitation. Similarly, the absence of a significant difference in PFS between subtypes across both treatment lines is consistent with the broader literature [13,14], reinforcing the notion that the probability of achieving disease control with currently available chemotherapy regimens does not differ substantially by KS subtype once systemic therapy is initiated.

### Limitations

This study has several inherent limitations. The retrospective design introduces potential selection bias, variability in treatment protocols across centers, and incomplete data capture in a proportion of patients. The small size of the AIDS-related KS subgroup (*n* = 10) substantially limits the statistical power of between-group comparisons and necessitates that all findings related to this group be interpreted as hypothesis-generating rather than definitive. A further limitation is that antiretroviral therapy (ART) regimens and CD4 cell count data could not be reliably and completely retrieved from retrospective records and were therefore not incorporated into the analysis. Progression analyses were considered exploratory in nature due to the limited number of events and high censoring rates.

Despite these limitations, the multicenter design, the geographic diversity of participating centers across multiple regions of Turkey, and the comparative analysis of two KS subtypes within a single cohort provide a level of representativeness not achievable in single-institution series and constitute the principal strengths of this study.

## 5. Conclusions

This multicenter real-world study demonstrates that classic and AIDS-related KS present with markedly distinct demographic and clinical profiles, with AIDS-related KS characterized by younger age, more advanced disease at diagnosis, and greater extracutaneous involvement. Despite these differences, response rates to initial treatment were high and comparable across both subtypes, underscoring the chemosensitivity of KS regardless of subtype. Overall survival was significantly longer in the classic KS group, reflecting the compounding burden of profound immunosuppression and advanced disease stage in AIDS-related KS rather than a fundamental difference in treatment responsiveness. The absence of a significant association between disease stage or lesion morphology and survival, combined with high rates of local recurrence across both subtypes, highlights the chronic and relapsing nature of KS and underscores the importance of long-term follow-up and individualized retreatment strategies. In particular, it should be noted that the findings regarding the AIDS-related Kaposi sarcoma subgroup are preliminary and hypothesis-generating due to the limited sample size. Multicenter prospective studies with standardized treatment protocols and systematic molecular profiling are needed to better define prognostic determinants and to optimize management strategies for both classic and AIDS-related KS in Mediterranean-endemic regions.

## Figures and Tables

**Table 1:** Comparison of demographic and clinical characteristics by subtype.

	Classic (*n* = 92)	AIDS-Related (*n* = 10)	Stat.	*p*
Age at diagnosis, median (range)	69 (19–89)	38.5 (23–64)	−4.447	<0.001 m
Sex			-	0.113 f
Female, *n* (%)	22 (23.9)	0 (0)		
Male, *n* (%)	70 (76.1)	10 (100)		
ECOG PS, *n* (%)			3.178	0.344 f
0	47 (51.1)	8 (80)		
1	39 (42.4)	2 (20)		
2	5 (5.4)	0 (0)		
Number of lesions, *n* (%)			11.155	0.004 f
0–10	72 (78.3) a	3 (30) b		
10–100	19 (20.7) a	6 (60) b		
>100	1 (1.1) a	1 (10) a		
Disease localization, *n* (%)			20.557	<0.001 f
Upper extremity	11 (12) a	0 (0) a		
Lower extremity	61 (66.3) a	2 (20) b		
Trunk	5 (5.4) a	0 (0) a		
Head and neck	1 (1.1) a	2 (20) b		
Multiple	11 (12) a	6 (60) b		
Clinical form, *n* (%)			1.519	0.549 f
Solitary	21 (28.4)	4 (44.4)		
Plaque	32 (43.2)	4 (44.4)		
Macule	21 (28.4)	1 (11.1)		
Extracutaneous involvement, *n* (%)			-	<0.001 f
No	84 (91.3)	3 (30)		
Yes	8 (8.7)	7 (70)		
Disease stage (classic type), *n* (%)				
Stage I-II	79 (85.9)	-		
Stage III	4 (4.3)	-		
Stage IV	9 (9.8)	-		
Disease stage (AIDS-related), *n* (%)				
Good risk	-	3 (30)		
Poor risk	-	7(70)		

m: Mann–Whitney U test, median (min.-max.); f: Fisher’s exact test, *n* (%); a,b: groups sharing the same letter do not differ significantly (Bonferroni-corrected Z-test); ECOG PS: Eastern Cooperative Oncology Group performance status.

**Table 2:** Treatment characteristics and response outcomes by subtype.

	Classic (*n* = 92)	AIDS-Related (*n* = 10)
Initial treatment, *n* (%)		
Observation	6 (6.5)	0 (0)
Surgical excision	23 (25)	0 (0)
Radiotherapy/Cryotherapy	32 (34.8)	2 (20)
Chemotherapy	19 (20.7)	7 (70)
Chemoradiotherapy	4 (4.3)	0 (0)
Radiotherapy + Excision	8 (8.7)	1 (10)
Best response to initial treatment, *n* (%)		
Complete response	78 (90.7)	8 (88.9)
Partial response	6 (7.0)	0 (0)
Progressive disease	2 (2.3)	1 (11.1)
Systemic therapy received, *n* (%)	35 (39.3)	8 (80)
First-line chemotherapy agent, *n* (%)		
Pegylated liposomal doxorubicin	9 (25.7)	7 (87.5)
Paclitaxel	15 (42.9)	1 (12.5)
Interferon	5 (14.3)	0 (0)
Vinblastine–bleomycin–adriamycin–dacarbazine	3 (8.6)	0 (0)
Vincristine	2 (5.7)	0 (0)
Etoposide	1 (2.9)	0 (0)
Best response to first-line chemotherapy, *n* (%)		
Complete response	17 (48.6)	6 (75.0)
Partial response	15 (42.9)	2 (25.0)
Stable disease	1 (2.9)	0 (0)
Progressive disease	2 (5.7)	0 (0)
Second-line chemotherapy agent, *n* (%)		
Pegylated liposomal doxorubicin	4 (33.3)	1 (50)
Oral etoposide	4 (33.3)	0 (0)
Paclitaxel	2 (16.7)	1 (50)
Vinblastine	1 (8.3)	0 (0)
Gemcitabine	1 (8.3)	0 (0)
Best response to second-line chemotherapy, *n* (%)		
Complete response	2 (16.7)	0 (0)
Partial response	7 (58.3)	0 (0)
Stable disease	1 (8.3)	1 (50)
Progressive disease	2 (16.7)	1 (50)

**Table 3:** Evaluation of chemotherapy types and progression status according to first-line treatment responses.

First-line Chemotherapy	SD	PR	CR	PD	No Progression	Progression
Classic						
PLD	1 (100)	2 (13.3)	6 (35.3)	0 (0)	6 (27.3)	3 (23.1)
Interferon	0 (0)	3 (20)	2 (11.8)	0 (0)	2 (9.1)	3 (23.1)
Paclitaxel	0 (0)	6 (40)	7 (41.2)	2 (100)	10 (45.5)	5 (38.5)
VBAD	0 (0)	1 (6.7)	2 (11.8)	0 (0)	3 (13.6)	0 (0)
Vincristine	0 (0)	2 (13.3)	0 (0)	0 (0)	1 (4.5)	1 (7.7)
Etoposide	0 (0)	1 (6.7)	0 (0)	0 (0)	0 (0)	1 (7.7)
AIDS-Related						
PLD	0 (0)	1 (50)	6 (100)	0 (0)	6 (100)	1 (50)
Paclitaxel	0 (0)	1 (50)	0 (0)	0 (0)	0 (0)	1 (50)

*n* (%); SD: Stable Disease; PR: Partial Response; CR: Complete Response; PD: Progressive Disease; PLD: Pegylated Liposomal Doxorubicin; VBAD: Vinblastine–Bleomycin–Adriamycin–Dacarbazine.

**Table 4:** Kaplan–Meier overall survival analysis.

	Mean (%95 CI)	Median (%95 CI)	Stat.	*p*
Subtype				
Classic	98.28 (76.21–120.34)	66.9 (39.9–93.9)	6.277	0.012
AIDS-related	41.81 (20.47–63.15)	22.67 (0–56.34)		
Clinical form				
Solitary	104.25 (56.37–152.14)	60.43 (11.88–108.98)	1.974	0.373
Plaque	70.42 (51–89.85)	57.77 (14.49–101.05)		
Macule	114.24 (53.43–175.05)	48.1 (0–111.65)		
Disease stage at diagnosis (AIDS-related)				
Good Risk	49.02 (3.03–95.01)	40.63 (0–84.48)	0.619	0.431
Poor Risk	38.72 (13.16–64.28)	22.67 (13–32.35)		
Disease stage at diagnosis (classic)				
Stage I-II	100.88 (76.83–124.93)	69.07 (42.03–96.11)	0.883	0.643
Stage III	43.81 (2.21–85.41)	20.17 (0–59.21)		
Stage IV	86.55 (26.57–146.52)	51.8 (3.1–100.5)		

**Table 5:** Univariate and multivariate Cox regression analysis of prognostic factors for overall survival in the classic group.

	Univariate HR (%95 CI)	*p*	Multivariate HR (%95 CI)	*p*
Age at diagnosis	1.018 (0.998–1.038)	0.076	1.022 (0.989–1.057)	0.197
ECOG (Ref.: 0)				
1	0.673 (0.402–1.126)	0.132	0.357 (0.116–1.098)	0.072
≥2	1.186 (0.420–3.355)	0.747	2.646 (0.428–16.350)	0.295
Comorbidity (Present vs. Absent)	1.211 (0.722–2.032)	0.468	—	
Smoking history (Ref.: None)				
Active smoker	1.072 (0.511–2.248)	0.854	0.622 (0.136–2.844)	0.541
Former smoker	1.459 (0.862–2.470)	0.160	1.702 (0.645–4.490)	0.282
Lesion count 10–100 (Ref.: 0–10)	1.350 (0.729–2.498)	0.340	—	
Clinical form (Ref.: Solitary)				
Plaque	1.505 (0.810–2.796)	0.196	0.938 (0.369–2.387)	0.893
Macule	1.062 (0.521–2.165)	0.868	0.694 (0.240–2.008)	0.500
Symptom (Ref.: None)	1.212 (0.731–2.012)	0.456	—	
Painful lesion (Ref.: None)	0.939 (0.526–1.678)	0.833	—	
Lymphedema (Ref.: None)	0.736 (0.230–2.357)	0.605	—	
Ulceration (Ref.: None)	1.008 (0.403–2.518)	0.986	—	
Extracutaneous involvement (Ref.: None)	1.198 (0.516–2.779)	0.674	—	
Hemoglobin	0.859 (0.750–0.984)	0.028	0.772 (0.636–0.936)	0.009
Neutrophil count	1.024 (0.882–1.190)	0.755	—	
Lymphocyte count	0.935 (0.621–1.409)	0.749	—	
Monocyte count	1.456 (0.430–4.929)	0.546	—	
Platelet count	0.998 (0.994–1.003)	0.506	—	
CRP	1.007 (0.970–1.045)	0.710	—	
Albumin	0.736 (0.418–1.296)	0.288	—	
LDH	0.993 (0.988–0.998)	0.003	0.988 (0.980–0.996)	0.003

HR: hazard ratio; CI: confidence interval; —: variable not included in the multivariate model. Statistically significant results in bold. Multivariate model: χ^2^ = 20.710, *p* = 0.014.

## Data Availability

The data presented in this study are available on request from the corresponding author.
